# Syphilis of the Throat and Nose—I

**Published:** 1908-05-02

**Authors:** 


### Laryngology and Rhinology.

### SYPHILIS OF THE THROAT AND NOSE.—I.

As in other parts of the body, the lesions of syphilis in the throat and nose may be divided into early and late forms; but in the upper respiratory tract, perhaps even more than elsewhere, the classical distinction between secondary and tertiary lesions is by no means sharply maintained. Deep and destructive inflammation may occur early in the course of the disease, and superficial patchy erythema—for instance, on the vocal cords—may ensue many years after infection. Above all, numerous cases are intractable to mercury or iodides separately, and yield only when both these drugs are given in combination. It is, therefore, preferable to speak of early and late forms as being less dogmatic than the classical nomenclature.

In the regions under discussion, a primary chancre occurs most frequently on the tonsil, fauces, and just within the nostril. The tonsil is the most common site, but it is altogether rare; indeed, it is rather to be wondered at that infection is not more frequent on the lips and mouth through drinking-vessels, spoons, and such like. There is an erosion with whitish, sticky base and surrounded by a sharply-defined margin; rarely is there much ulceration; and the typical induration is found on palpation. The amount of swelling around the sore is most variable: sometimes it is enormous, in other cases so slight as to escape attention. An unnoticed extra-genital chancre is, perhaps, commoner than is generally believed. There is usually but little pain. The submaxillary lymphatic glands are always enlarged in tonsillar cases, and usually also those in the parotid region when the chancre is in the nose. The hard, indolent bubo which results is very typical. Extra-genital chancres are liable to be followed by very severe constitutional symptoms.

These give rise to but slight symptoms. Unless specially looked for, they generally escape attention and often closely resemble the effects of simple catarrh, unless the resulting redness is markedly patchy or unless definite mucous patches are present. In the nose, redness and swelling of the lining membrane may be seen; mucous patches are occasionally found, especially about the vestibule, but are decidedly uncommon. On the fauces the lesions are more often seen, and are frequently quite characteristic. The mildest takes the form of a symmetrical hyperæmia of a peculiar dusky colour, which can only be described as a blend of the normal red of the parts with the coppery tint of syphilitic eruptions. This occupies the soft palate and faucial pillars, and is limited in front by a sharply-defined circinate border which should always arouse suspicion. In other cases the hyperæmia is less extensive and is confined to a few irregular blotches with sharply marked edges. Mucous patches appear as smooth or slightly raised whitish patches with serpiginous margins surrounded by hyperæmia; the term "snail-track" vividly describes their characteristic appearance. They occur on the tonsils, fauces, posterior pharyngeal wall and both surfaces of the palate. The spot where the arch of the palate joins the base of the uvula on either side is a favourite seat for these early lesions.

In the larynx the early lesions are not often looked for; an erythema, more patchy than that of simple catarrh, may be seen. Condylomata occur, especially on the lingual surface of the epiglottis; they are rare on the cords; the writer has seen a pair of minute symmetrical condylomata on the cords of a phthisical patient, and they healed rapidly under mercury. These early lesions in the fauces or larynx are accompanied by indolent swelling of the cervical glands.

The infiltration may be diffuse or circumscribed, the latter being synonymous with gumma; and in either case ulceration, superficial or deep, may ensue. The resulting lesions are extraordinarily diverse and may closely imitate every variety of disease to which the parts are liable. Only the more charactcrisitc appearances can be described here.

Extensive ulceration is common in the nose, and is often accompanied by necrosis. The discharge is usually plentiful, and tends to dry into green and black crusts. The affection can be readily distinguished from atrophic rhinitis for, when the crusts are removed, the mucous membrane is seen to be thickened, red, and inflamed, ulceration is present, and bare or necrosed bone may be found. The disease is usually, but not always, bilateral. Ulceration and necrosis may affect the turbinals, or indeed any part of the nose, but is most marked on the septum. Here the triangular cartilage is commonly perforated, but this is also a result of lupus, tuberculosis, and the so-called "idiopathic" perforation; in syphilis, however, the perforation generally involves the bony septum as well, and this is practically pathognomonic. In neglected cases of necrosis the odour is peculiar and very horrible. After severe ulceration the resulting fibrosis causes the nose to sink in; a sharp indentation of the profile immediately below the nasal bones is the commonest deformity and differs from the flat bridge of congenital syphilis. Perforation of the ala nasi sometimes occurs, but less often than in lupus; destruction of the columella is more common and produces a peculiar depression of the tip of the nose. Scarring at the vestibule may cause the most extreme stenosis of the anterior nares.

A rarer form of nasal syphilis is localised gumma of the septum. This appears as a globular swelling of the septum projecting into both nares, and if untreated breaks down into a perforation. In appearance it closely resembles an abscess or a hæmatoma. Sometimes, without visible ulceration, the entire mucous membrane becomes so swollen as to completely occlude the nares; this condition is accompanied by a profuse ichorous discharge, and is not readily recognised as syphilitic.

